# Use of the Hippocratic or other professional oaths in UK medical schools in 2017: practice, perception of benefit and principlism

**DOI:** 10.1186/s13104-017-3114-7

**Published:** 2017-12-29

**Authors:** Ben Green

**Affiliations:** 1 Institute of Medicine, University Centre Shrewsbury, Guildhall, Shrewsbury, SY3 8HQ UK; 2 The Medical School, University of Chester, Parkgate Road, Chester, CH1 4BJ UK

**Keywords:** Hippocratic Oath, Professionalism, Medical ethics, Patient safety, Principlism, Ethics, Professional codes, Medical practice

## Abstract

**Objective:**

This paper concerns the continued use of the Hippocratic Oath in United Kingdom (UK) medical schools. A survey of all UK medical schools looked at which schools use the Oath, which variants they use, and what they perceive to be the benefits of using the Oath. 27 schools participated in the study.

**Results:**

Although some authors have deemed the Oath as out of date for the purposes of modern medicine [1], new variants of the Oath have been embraced and 19/27 (70%) of schools use an Oath, with some Universities asking student doctors to acknowledge this Oath on entry to and graduation from medical school. There is a renewed interest in use of the Oath, with use in some Schools on admission and graduation. Reasons for adopting the Oath include a desire to enhance good practice and to prevent unwanted behaviour. Variants of the Oath used were analysed according to which bioethical principles are contained within them and some do not accord with all principles. A new variant of the Oath is proposed which encompasses all four bioethical principles.

**Electronic supplementary material:**

The online version of this article (10.1186/s13104-017-3114-7) contains supplementary material, which is available to authorized users.

## Introduction

The Hippocratic Oath has been a feature of medical study and practice for hundreds of years, across the globe [2]. In recent decades its continued use has been criticised as the Oath has been deemed out of date for the purposes of modern medicine [1], with some favouring the use of regulatory codes provided by bodies such as the UK General Medical Council (GMC)’s *Good Medical Practice* [3].

Many medical schools feature the Oath in a revised form, such as the twentieth century version written by Louis Lasagna (1923–2003) (please see Additional file 1: Appendix S1) [4]. Lasagna updated the Oath in various ways, removing references to gods, prohibitions on surgery and abortion, and descriptions of fees and how students should relate to their teachers [5].

This paper establishes the current use of the Oath in UK medical schools, the version of the Oath used by such schools and their perception of the purpose or benefit of the Oath. There is analysis of the different Oaths used, based on the four bioethical principles—beneficence, non-malificence, autonomy, and social justice [6].

The author is the Director for Undergraduate Medicine at a University with a new medical school in development, and an interest in the current status of the Oath in developing that School.

## Main text

The study was considered by the ethical committee of the Institute of Medicine with Approval Number 1172/16/BG/IoM.

### Methods

We sought responses from all 32 UK medical schools using a structured questionnaire, which asked:

1. Does your medical school require students to take an Oath on graduation?

If yes, how is this administered? (e.g. verbally at graduation).

If so, which version of the Hippocratic Oath do you use?

2. What do you consider are the benefits of using the Oath (or not using the Oath)?

The questionnaire was developed in discussion with the Faculty Research Ethics Committee. Questionnaires were sent to the Deans of all UK medical schools, with a stamped return envelope and the request was followed up twice more; at 1 and 2 months. Responses were analysed using a mixed methods approach including descriptive statistics and content analysis. The Oaths were analysed against the four bioethical principles. Content analysis used open questions i to generate free text to identify themes generated by the participants and coded by the author [7]. Qualitative analysis considered the approach set out in the ‘Consolidated criteria for reporting qualitative research’ (COREQ) [8].

### Results

Twenty-Seven schools responded. Of these 19 required graduands to say a version of the Hippocratic Oath at graduation, and three invited graduands to say a version of the oath, but did not absolutely require this. Only three schools did not require graduands to say an Oath or any variant. One school, St. Andrews, did not require the oath as their students graduate from a 3 year course, and go on to do clinical training elsewhere. The response rate of 84% was satisfactory. Reasons for non-response might include pressure of other business.

A few schools noted that the Oath had been re-introduced at the request of students, e.g. East Anglia, and Southampton. Several schools noted that they had consulted with students about the version of the Oath used, e.g. Hull and York.

Of the three schools not using an oath, Cambridge did not think the swearing of an oath ‘appropriate’, and noted that their graduating students affirmed the GMC ‘Duties of a Doctor’ at a Declaration Ceremony. Kings College London also referred to its adherence to the GMC ‘Duties of a Doctor’, but said it was consulting with students on the use of an Oath.

#### Versions of the Oath used

The Schools all used modern variations of the Hippocratic Oath or close approximations. The most frequently mentioned variant was the World Medical Association Declaration of Geneva, used by Warwick, Hull and York, Liverpool, Leicester, and East Anglia. A summary of the Oaths required in 19 medical schools is given in Table 1.

**Table 1:** Variants of the Hippocratic Oath required by 19 medical schools

Oath used	Numbers of school requiring
Their own variant, e.g. based on Lasagna	8
Declaration of Geneva	7
A statement based on the GMC code of good medical practice	3
Original Lasagna Oath	1

Various schools had adapted or written a version themselves, e.g. Aberdeen uses an Oath written by Professor Eric Matthews, who taught Philosophy of Medicine there. Some schools employed a similar ethical oath across all graduating health care practitioners, e.g. Sheffield and Birmingham.

Bristol uses a version written by a previous professor of Medical Ethics, covering all four themes of biomedical ethics.

Glasgow has used an amended version since 2003 [9].

Edinburgh’s graduation oath has been used since 2004 (Reproduced in Additional file 1: Appendix S2) and mentions all four themes of biomedical ethics. Queen’s University Belfast’s version is called the *Sponsio Academica* and similarly mentions the four themes of biomedical ethics.

Swansea’s graduation oath was adapted by Professor Julian Hopkin, the first Dean, and adheres to two themes of biomedical ethics: beneficence, and social justice.

One school, Plymouth, specifically noted its version was drafted by students.

#### How the oath is administered

Most schools use verbal recitation of the oath at graduation, e.g. school such as Liverpool, Leicester, Warwick, and Cardiff.

Swansea has two students read out their oath, one in English and one in Welsh, with the whole cohort replying ‘I will’ to the English version and ‘Gwnaf’ to the Welsh.

The oath is also re-affirmed by clinical academics and postgraduates at some ceremonies, e.g. Liverpool.

Queen’s University Belfast’s *Sponsio Academica* oath is at the graduation ceremony and where students are unable to attend the ceremony they later meet with a senior clinical academic and recite the *Sponsio* in their presence.

East Anglia introduced the use of an Oath in 2015 at the suggestion of students. Students read the Oath once assembled for their class graduation photograph.

Queen Mary University of London’s incorporates the Declaration of Geneva into an annual ‘Rites of Passage’ held at St. Paul’s Cathedral.

A few medical schools noted that an oath was employed twice—on beginning medical studies and also at graduation, e.g. Bristol.

#### Perceived benefits of the Oath

Themes identified in Schools’ reasons for using the Oath are summarised in Table 2.

**Table 2:** Themed reasons given by schools for requiring the Oath

Reason	Number of schools expressing this reason
To signify transition from student to professional (entry into a profession)	7
Tradition for medicine	7
Professional values affecting patient care	5
To add gravitas into a ceremony	4
Oath valued by students	4
Oath valued by family	3
Promotes cohesion in profession	1

Professor Kumar, Dean of Warwick Medical School noted that the taking of the Oath signalled an entry into a profession. This reason was echoed by several Schools.

Professor McKeown of Queen’s University Belfast referred to the ‘profound effect which reciting this Oath in public has on them in relation to the realisation they are entering a noble and trusted profession which places patients at the centre of their working lives’.

Dr. Riley of Cardiff described the Oath as a ‘focal point of the graduation ceremony’ and noted that the prospect of taking the Oath was ‘used during the final year of study to align the student’s perceptions of patient centred care’.

Professor Frenneaux of East Anglia noted that taking the Oath ‘added a sense of connection to a cohort bound by a solemn pledge’. Several schools noted the Oath distinguished medical graduation ceremonies from other subjects.

Dr. Joynes, of Liverpool noted that it was a reminder of responsibilities and privileges associated with the profession and how graduands had a ‘great honour in looking after the lives of others’.

It was noted by one School that despite supporting the Oath at graduation, this was a voluntary endeavour on behalf of students and that in the UK a doctor the only ‘binding professional code for medical practitioners is Good Medical Practice and associated documents’.

### Discussion

#### Increase in use of the Oath

Hurwitz and Richardson reported that in 1997 only 50% of UK medical schools used a form of the Hippocratic Oath whereas in 2017 this study found that 19/27 (70%) of schools required an Oath, indicating an increase in the use of the Oath in the last 20 years [10]. Comparing the UK to the USA, 100% of US schools in a sample of 67 employed the Oath [11].

Some UK schools use the Oath twice, on entry to medical school and on graduation. 88% of US medical schools adopt this multiple use of the oath [11].

#### Professionalism, codes of behaviour versus bioethical principles

Some authors placed the responsibility for advice on doctors’ conduct and behaviour with regulatory authorities, rather than with the individual practitioner. In 2003 Colvin stated ‘it is the responsibility of the General Medical Council in the UK and similar licensing authorities elsewhere to give clear and unambiguous advice on the conduct expected of a doctor’, and placed an emphasis on ‘sanctions’ and ‘performance procedures’ to ensure compliance [12].

The text of GMC guidance, *Good Medical Practice* (2013), mentions the word principles three times, but never elaborates on what these principles are, and does not mention the words ‘ethics’, ‘ethical’, or ‘bioethical’ once. Exclusion of such terms is a significant omission [3].

The Oath could be said to enshrine bioethical principles, although principlism as a defined concept was probably not in the mind of the first doctors to espouse the Oath’s use. Not all versions of the oath in current use align to all four principles. Without identifying desirable underlying ethical principles it could be that codes of behaviour for healthcare professionals drift from these principles.

It could be argued that codes of behaviour [13] do not always meet individual circumstances or apply in changing times and that, without an individual reflecting and relating their own decisions and actions to clear and well understood ethical principles, anomalous or undesirable prejudices or actions may follow. Adherence to underlying ethical principles, enshrined in some form of the Oath would seem to place an emphasis on personal responsibility for medical conduct, rather than projecting sole reliance on an external regulatory body.

#### Evolution of the Oath—promoting a dynamic Oath

Few UK medical schools use the original version of the Oath, whereas reportedly 43% of United States (US) schools still use this [11]. Twentieth century revisions predominate across the UK and US (please see Table 1).

Even so, twentieth century versions of the Oath have not aged well, accumulating criticisms over time, like barnacles. Shmerling noted that there was a need to incorporate patient’s preferences, the role of adequate information to make decisions, to curtail profiteering and avoid conflicts of interest, protect patients in clinical trials, promote equality of treatment, and recognise the imperative to not practice whilst functionally impaired, e.g. through illness [14].

That a professional oath may change over time is clear from some work, which would seem to indicate that not all oaths specifically prohibit doctor-patient sexual relationships [15]. Clearly society can vary the contents of oaths or codes to suit. That an oath should be based upon, and reinforce, bioethical principles would seem to be even more relevant in light of societal re-writing of oaths or codes. Any argument from first principles might guide the practitioner away from intimate relationships with patients on the basis that there is an imperative for non-malificence, and that the transgression of sexual boundaries carries a significant potential for harming the patient. Laudable though the taking of an oath might be, if coupled with an ethical training based upon principlism, an oath would provide additional protection for patients and society.

## Conclusion

The majority of UK medical schools require or invite their students to say a variant of the Hippocratic Oath at graduation, with a few schools also using a variant at the outset of their medical studies. In this study only three schools did not use a form of the Hippocratic Oath and one of these was considering introducing a variant in the near future. Use of the Oath seems to be flourishing as schools report it is both popular with students and the public (please see Table 2). Not all variants of the Oath cover all four bioethical principles and a new variant of the Oath is proposed (Additional file 1: Appendix S3) which aims to embrace them all.

## Limitations

84% of UK medical schools (27/32 schools) responded and so we lack data from five schools.

## Additional files

**Additional file 1: Appendix S1.** Hippocratic oath: Lasagna version.

**Additional file 2: Appendix S2.** Edinburgh Medical Oath.

**Additional file 3: Appendix S3.** Revised Medical Oath Based Upon Principlism.

## Abbreviations

COREQ: consolidated criteria for reporting qualitative research
GMC: General Medical Council
UK: United Kingdom
US: United States
