# Supplementary material for: Use of the Hippocratic or other professional oaths in UK medical schools in 2017: practice, perception of benefit and principlism
Source: BMC Res Notes. 2017 Dec 29;10:777. doi: 10.1186/s13104-017-3114-7 (PMC5747024; doi:10.1186/s13104-017-3114-7)
Supplement: Supplementary file 1 — Additional file 1: Appendix S1. Hippocratic oath: Lasagna version. [file 13104_2017_3114_MOESM1_ESM.docx]

Appendix S1: Hippocratic oath: Lasagna version

I swear to fulfill, to the best of my ability and judgment, this covenant:

I will respect the hard-won scientific gains of those physicians in whose steps I walk, and gladly share such knowledge as is mine with those who are to follow.

I will apply, for the benefit of the sick, all measures [that] are required, avoiding those twin traps of overtreatment and therapeutic nihilism.

I will remember that there is art to medicine as well as science, and that warmth, sympathy, and understanding may outweigh the surgeon's knife or the chemist's drug.

I will not be ashamed to say "I know not," nor will I fail to call in my colleagues when the skills of another are needed for a patient's recovery.

I will respect the privacy of my patients, for their problems are not disclosed to me that the world may know. Most especially must I tread with care in matters of life and death. If it is given me to save a life, all thanks. But it may also be within my power to take a life; this awesome responsibility must be faced with great humbleness and awareness of my own frailty. Above all, I must not play at God.

I will remember that I do not treat a fever chart, a cancerous growth, but a sick human being, whose illness may affect the person's family and economic stability. My responsibility includes these related problems, if I am to care adequately for the sick.

I will prevent disease whenever I can, for prevention is preferable to cure.

I will remember that I remain a member of society, with special obligations to all my fellow human beings, those sound of mind and body as well as the infirm.

If I do not violate this oath, may I enjoy life and art, respected while I live and remembered with affection thereafter. May I always act so as to preserve the finest traditions of my calling and may I long experience the joy of healing those who seek my help.
