# Supplementary material for: Use of the Hippocratic or other professional oaths in UK medical schools in 2017: practice, perception of benefit and principlism
Source: BMC Res Notes. 2017 Dec 29;10:777. doi: 10.1186/s13104-017-3114-7 (PMC5747024; doi:10.1186/s13104-017-3114-7)
Supplement: Supplementary file 2 — Additional file 2: Appendix S2. Edinburgh Medical Oath. [file 13104_2017_3114_MOESM2_ESM.docx]

Appendix S2: Edinburgh Medical Oath

I declare that I will practise my profession to the best of my knowledge and ability, in good conscience and with integrity.

In my practice the care of my patients will be my first consideration.

I will strive to prevent and treat disease, improve quality of life, provide support in times of suffering.

I will respect the autonomy, confidences and dignity of all my patients in their living and in their dying.

I will promote the health and welfare of the community.

I will treat with respect my colleagues and all who contribute to the well being of my patients.

I will constantly seek to gain in knowledge and understanding, and to pass on the art and science of medicine to others, as my teachers have done before me.

I will treat all patients equally and without prejudice.

I will not breach these obligations, or abuse the trust placed in me, either under threat or for personal gain.

I make this declaration solemnly, freely, and upon my honour.
