# Supplementary material for: Use of the Hippocratic or other professional oaths in UK medical schools in 2017: practice, perception of benefit and principlism
Source: BMC Res Notes. 2017 Dec 29;10:777. doi: 10.1186/s13104-017-3114-7 (PMC5747024; doi:10.1186/s13104-017-3114-7)
Supplement: Supplementary file 3 — Additional file 3: Appendix S3. Revised Medical Oath Based Upon Principlism. [file 13104_2017_3114_MOESM3_ESM.docx]

Appendix S3: Revised Medical Oath Based Upon Principlism

I declare that, as a foundation of my actions, I will practise my profession to the best of my knowledge, ability and insight, in good conscience and with probity, treating all people equally and fairly, without prejudice.

I will always remember my position of power and trust, and hold myself accountable for my actions and their consequences, eschewing recklessness.

I will respect the autonomy, confidences and dignity of all my patients in their living and in their dying.

In my practice the care and treatment of patients will be my first consideration. I will always seek to improve and maintain my patients’ health and strive to cause no deliberate or negligent harm to my patients or others.

I will strive to prevent and treat disease, improve quality of life, provide support in times of suffering and promote and protect the health and wellbeing of the communities that I live and work in.

I will treat my colleagues and all who contribute to the well being of my patients with respect.

I will continue to seek knowledge, understanding, and insight, to improve my clinical skills and to teach the art and science of medicine to others, as my teachers have done before me.

I will not breach these obligations, or abuse the trust placed in me, either under threat or for personal gain.

I make this declaration solemnly, freely, and in good faith.
